# Perceptions in the management of colorectal peritoneal metastases: A bi-national survey of colorectal surgeons

**DOI:** 10.1515/pp-2019-0022

**Published:** 2019-10-30

**Authors:** Vignesh Narasimhan, Satish Warrier, Michael Michael, Jacob McCormick, Robert Ramsay, Craig Lynch, Alexander Heriot

**Affiliations:** Department of Surgery, Peter MacCallum Cancer Centre, Melbourne, Australia

**Keywords:** colorectal peritoneal metastases, cytoreductive surgery, hyperthermic intraperitoneal chemotherapy, peritoneal carcinomatosis

## Abstract

**Background:**

There is great variability in the uptake of cytoreductive surgery (CRS) with hyperthermic intraperitoneal chemotherapy (HIPEC) in the management of colorectal peritoneal metastases (CRPM) in Australia and New Zealand. This study aims to provide a snapshot of perceptions among colorectal surgeons in the management of CRPM.

**Methods:**

A structured ten-question online survey was sent to all colorectal surgeons, with three questions on clinical experience and demographics, one on health economics and six on hypothetical clinical scenarios. Scores were collated and reported based on Likert scales.

**Results:**

Eighty-one respondents (36.2%) completed the survey. Most surgeons (66.7%) strongly disagreed with offering CRS and HIPEC at all hospitals. The majority (87.7%) agreed that CRS and HIPEC offered a higher survival benefit than systemic chemotherapy in pseudomyxoma peritonei (PMP), and 69.1% in CRPM (comparators: 60.5% ovarian cancer, 14.8% gastric cancer). There were mixed strategies in managing low-volume, isolated peritoneal recurrences. The majority did not recommend second-look laparoscopy, but favoured operative management of Krukenberg tumours. In the presence of incidental peritoneal metastases, only 29.6% favoured biopsy only and referring the patient to a peritoneal disease centre.

**Conclusions:**

Response rate was relatively low. In Australia and New Zealand, colorectal surgeons see a strong role for CRS and HIPEC in the management of PMP and CRPM. The role of “second look” surgery in high-risk cases is controversial and not supported. Krukenberg tumours are viewed as surgical disease. Regular updates and collaboration with peritoneal centres may help surgeons stay abreast with latest evidence in the field.

## Introduction

Peritoneal metastases confer the worst survival among patients with metastatic colorectal cancer (CRC) [1]. Historically, patients with peritoneal metastases had an overall survival of only 6–9 months [2, 3]. The greatest advance in the management of peritoneal metastases has been the advent and adoption of cytoreductive surgery (CRS) with hyperthermic intraperitoneal chemotherapy (HIPEC). Verwaal et al. [4] demonstrated in a randomised trial that CRS and HIPEC offers an improved survival of 22.3 months compared to 12.6 months with systemic chemotherapy only in the management of colorectal peritoneal metastases. Since then, a number of other studies have reported favourable median survival of 30–58 months with CRS and HIPEC, with a 27–46% 5-year survival [5, 6, 7, 8, 9, 10, 11].

Despite mounting evidence in favour of CRS and HIPEC, there remains ongoing skepticism about its role and efficacy among medical oncologists and surgeons alike. This is reflected in differing approaches to peritoneal disease by different hospitals and clinicians, as well as varying guidelines in management. However, it is unclear whether skepticism is due to lack of awareness and knowledge among clinicians in the management of peritoneal disease. Recent studies have shown that poor awareness of the value of the CRS and HIPEC in the management of colorectal peritoneal metastases (CRPM) contributes to reduced utilisation of CRS and HIPEC [12, 13].

In Australia and New Zealand, management of peritoneal disease is restricted to selected centres, with most peritoneal units comprised of colorectal surgeons. This study aimed to gain a snapshot of perceptions in the management of peritoneal metastases from colorectal cancer among colorectal surgeons in Australia and New Zealand.

## Materials and methods

A structured ten-question online Survey Monkey survey was sent to all colorectal surgeons affiliated with the Colorectal Surgical Society of Australia and New Zealand (CSSANZ). Three questions were based on clinical experience and demographics, one on health economics and the remaining six were hypothetical scenarios evaluating management of peritoneal disease. These six scenarios were a range of classical cases involving management of isolated peritoneal metastases, synchronous and metachronous peritoneal disease, high-risk patients and risk factors for peritoneal recurrence. The survey design ensured that surveys could only be returned if all questions were completed.

After the survey was sent out, a reminder was sent to those who had not responded 2 weeks later. The survey was open for 1 month. Survey questions are presented as Supplementary Data 1.

### Statistical analyses

Data were collated and analysed using Microsoft Excel. Bar graphs were created to demonstrate the percentage of respondents choosing each management option. *χ*^2^-Test was used to compare differences in responses based on surgical experience.

### Ethics

This project was approved by the Institutional Research Ethical Review Committee (LNR/19/PMCC/25).

## Results

Surveys were sent to 224 colorectal surgeons affiliated with CSSANZ. Eighty-one (36.2%) surveys were successfully completed. Nineteen (23.5%) respondents were from New Zealand, with the remaining 62 (76.5%) being Australian colorectal surgeons.

Scores were collated based on classical Likert scales into five categories and reported accordingly.

### Demographics and experience

The majority (88.9%) of colorectal surgeons were practicing in metropolitan cities. Of the 81 respondents, over two thirds (67.9%) had 5 or more years experience as a colorectal surgeon.

### Health economics

**Figure 1:**
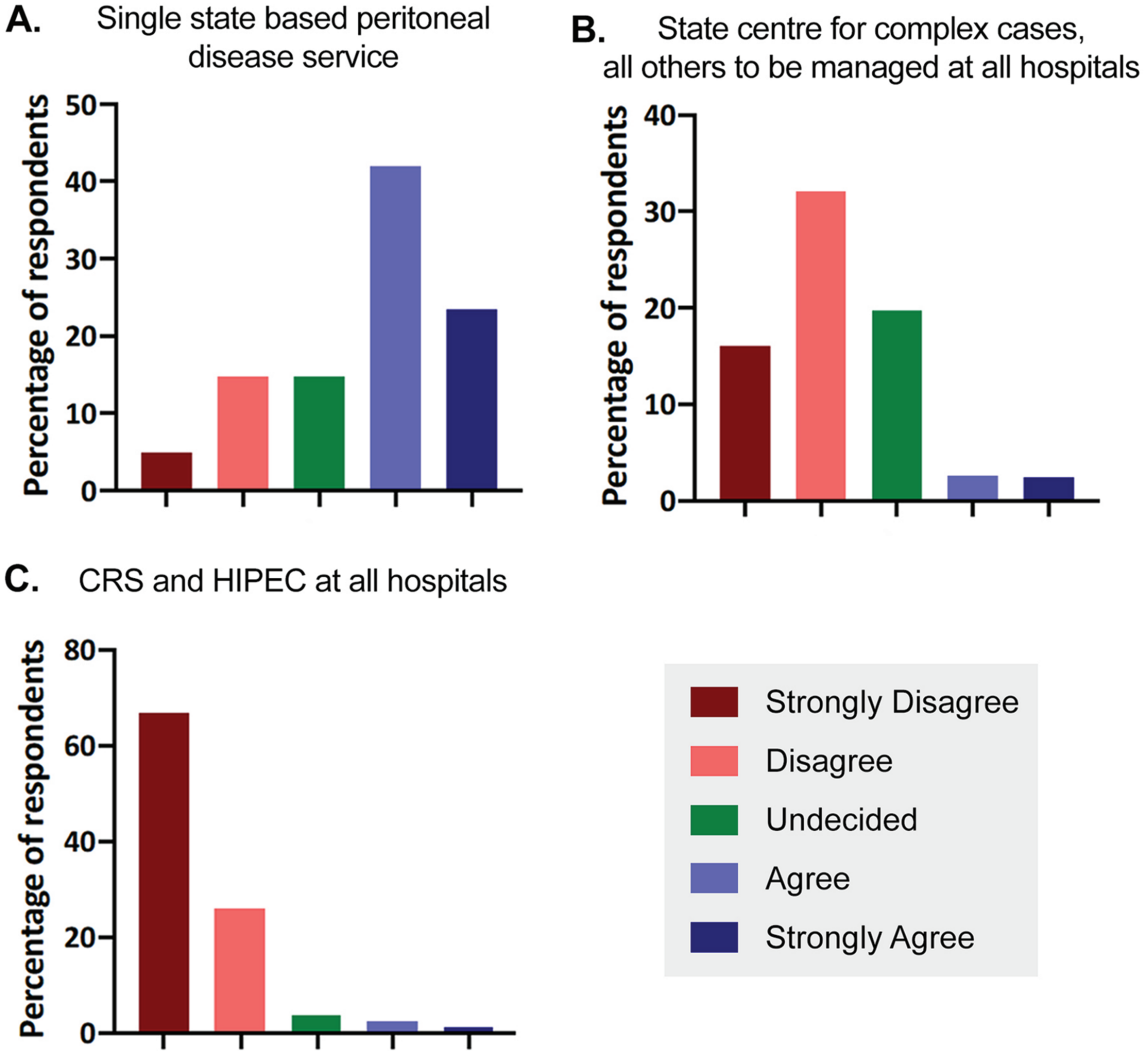
Models of care for treating peritoneal disease.

The majority (65.4%) of surgeons either agreed or strongly agreed with the existing model of a single state based service being the most effective (Figure 1A). The majority (48.1%) were not in favour of managing only complex cases at a state centre (Figure 1B). Most surgeons (66.7%) strongly disagreed with offering CRS and HIPEC at all hospitals (Figure 1C).

(A) Most surgeons agree with the existing model of a single state based peritoneal centre. (B) The majority disagree with the concept of only complex cases being managed at a state centre. (C) Almost all surgeons disagree with the concept of offering CRS and HIPEC at all hospitals.

### Role of CRS and HIPEC over systemic chemotherapy

**Figure 2:**
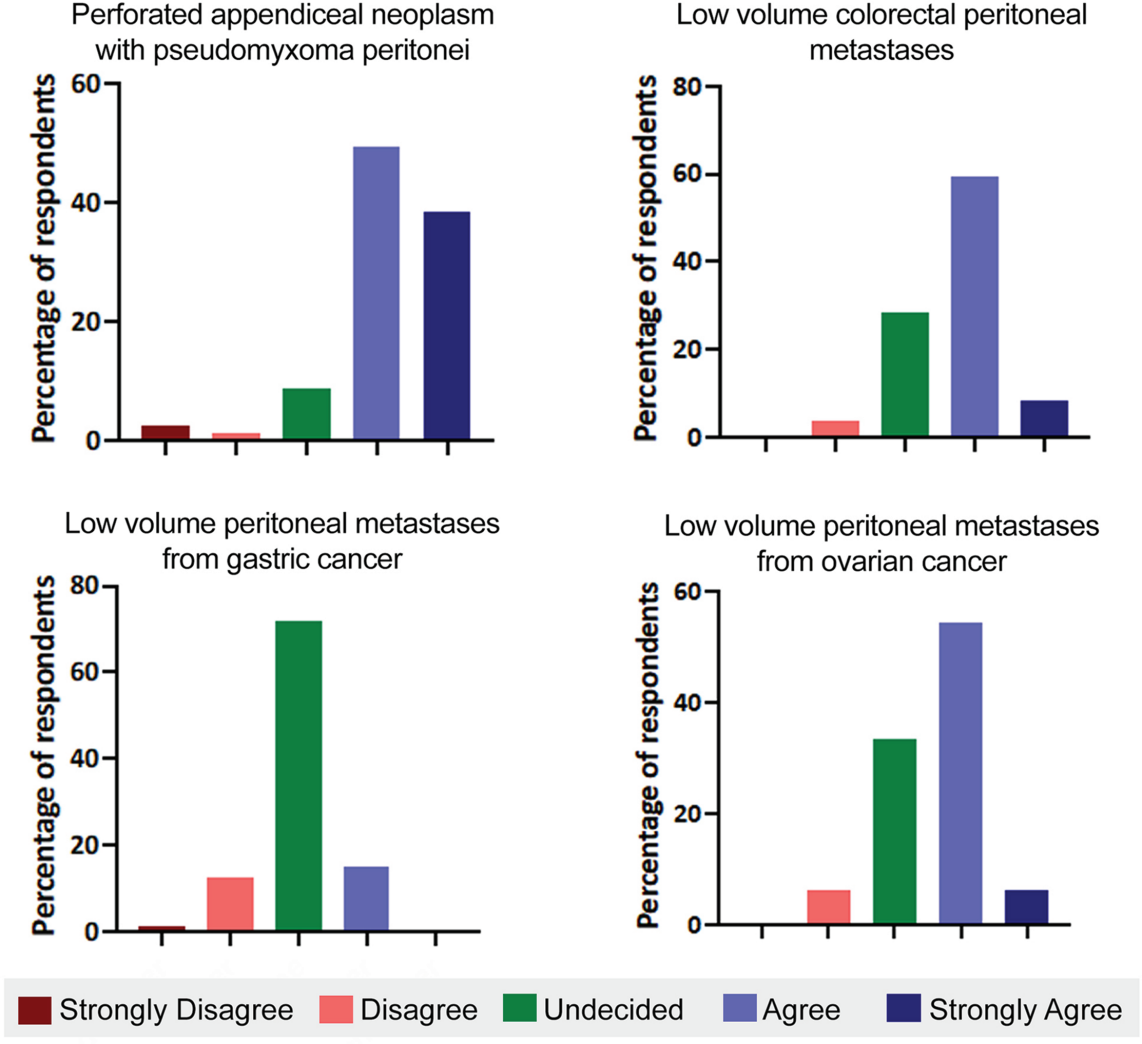
Improvement in survival with CRS and HIPEC compared to systemic chemotherapy in different situations.

In treating pseudomyxoma peritonei from perforated appendiceal neoplasms, 87.7% of surgeons agreed or strongly agreed that CRS and HIPEC offered a higher survival benefit than systemic chemotherapy (Figure 2A). Comparatively, with CRPM, only 69.1% felt CRS and HIPEC offered improved survival over systemic chemotherapy. Notably, over a quarter of surgeons (27.1%) felt there was no difference in survival offered by CRS and HIPEC (Figure 2B). In gastric cancer, most (70.4%) felt there was no benefit from CRS and HIPEC over systemic chemotherapy (Figure 2C), while in ovarian cancer, 60.5% felt CRS and HIPEC offered an improved survival (Figure 2D).

(A) Pseudomyxoma peritonei from perforated appendiceal neoplasms; (B) low volume colorectal peritoneal metastases; (C) low volume gastric cancer peritoneal metastases; and (D) low volume peritoneal metastases from ovarian cancer.

### Management of imaging detected isolated low volume peritoneal recurrence

**Figure 3:**
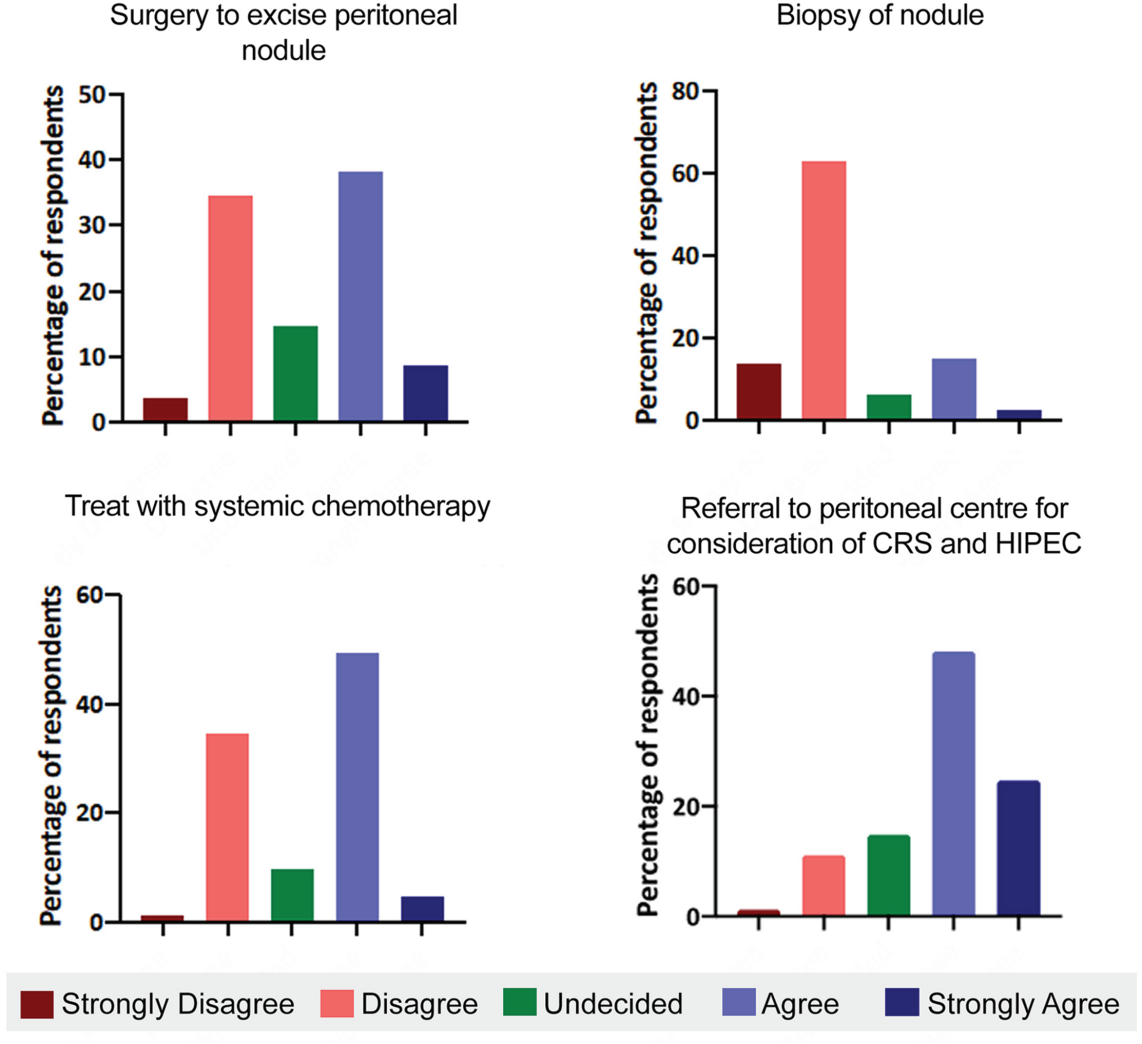
(A–D) Responses to different options in the management of an isolated 2 cm peritoneal recurrence.

Question 6 explored the management of an imaging detected 2 cm isolated peritoneal recurrence, 18 months after primary colorectal cancer resection. There were mixed strategies among surgeons in managing this, with 45.7% favouring surgery to excise the isolated nodule (Figure 3A). Over three quarters (75.3%) disagreed or strongly disagreed with biopsing the nodule (Figure 3B). Over half (58.1%) favoured systemic therapy for treating low volume isolated peritoneal disease (Figure 3C). Almost three quarters (72.8%) agreed or strongly agreed with referring such a case for consideration of CRS and HIPEC (Figure 3D).

### Role of pro-active ‘early relook’ surgery

**Figure 4:**
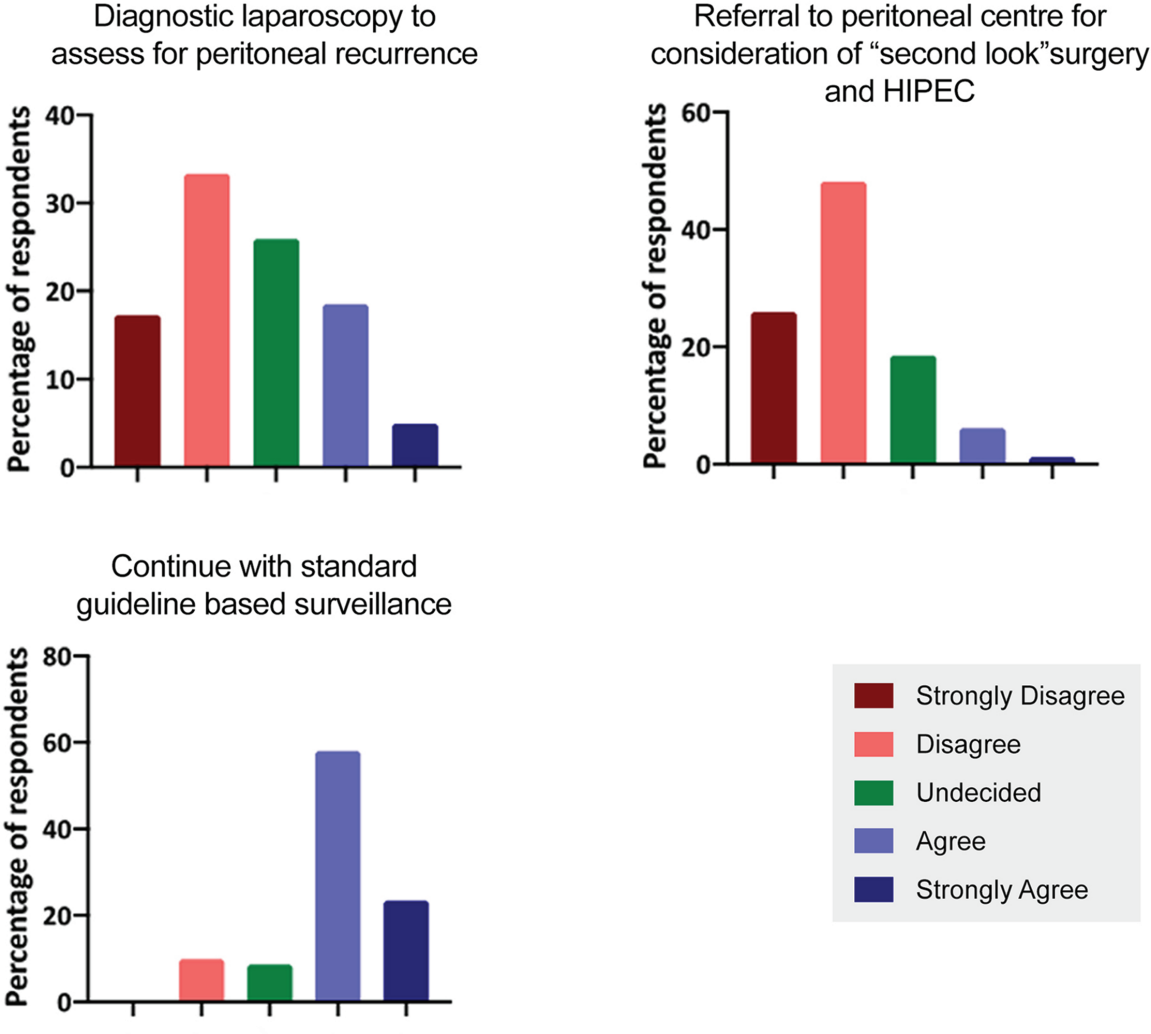
(A–C) Responses to options in managing a high-risk case for peritoneal recurrence with no imaging evidence of peritoneal recurrence.

Question 7 evaluated the role of a pro-active “early relook” in a high-risk case of a T4a tumour with no imaging evidence of peritoneal recurrence. The majority (49.4%) of surgeons disagreed or strongly disagreed with offering a diagnostic laparoscopy at 6 months (Figure 4A). Almost three quarters (72.3%) would not refer this case to a peritoneal centre for consideration of “early relook” and HIPEC (Figure 4B), with the majority of surgeons (81.5%) opting for standard national guideline based surveillance (Figure 4C).

### Management of Krukenberg tumours

**Figure 5:**
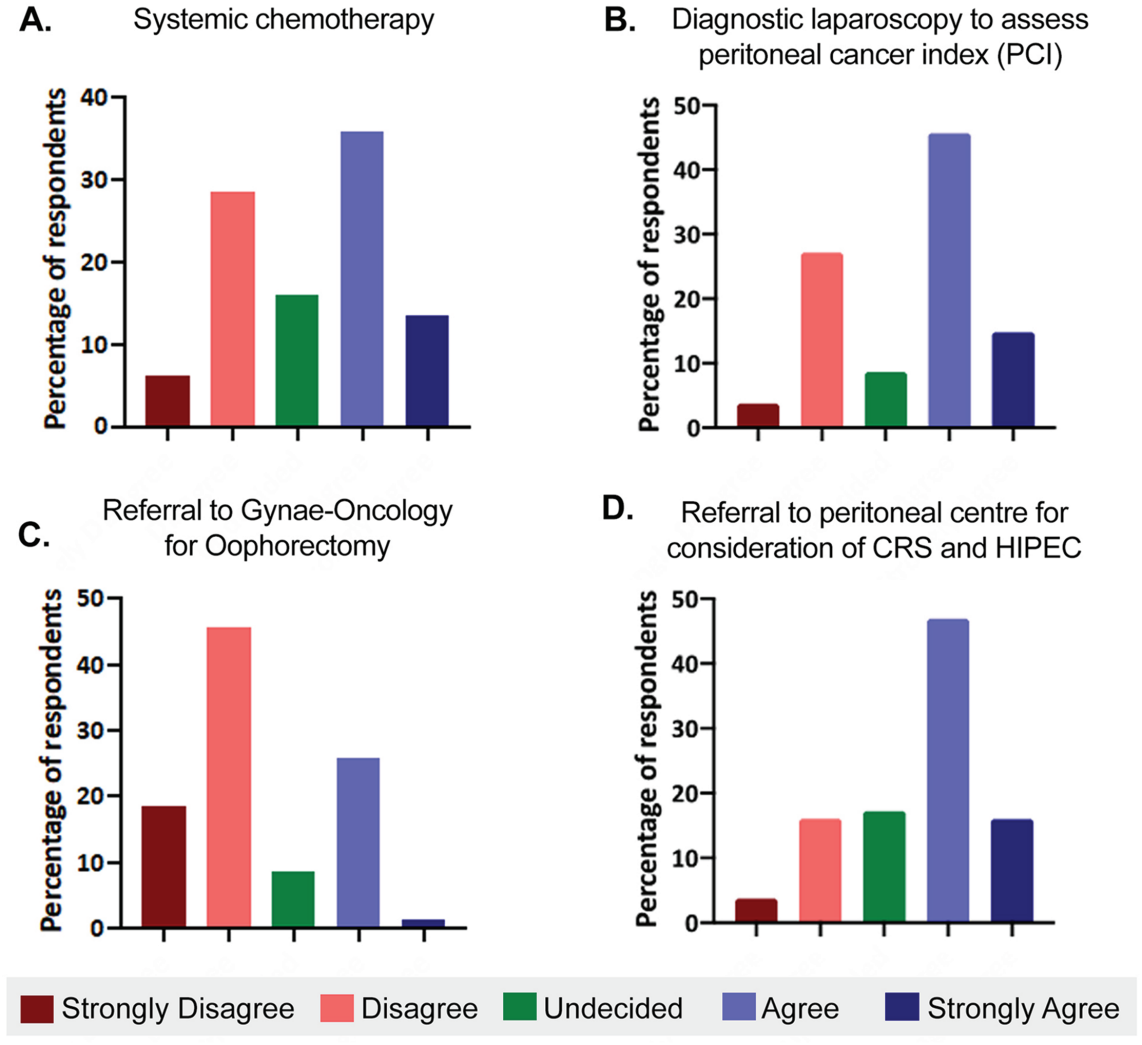
(A–D) Responses to options in managing an isolated left Krukenberg tumour.

Question 8 evaluated responses to an imaging detected isolated left Krukenberg tumour 9 months after primary colorectal tumour resection. Almost half (49.4%) agreed or strongly agreed to treat this case with systemic chemotherapy (Figure 5A). The majority (60.4%) agreed or strongly agreed with performing a diagnostic laparoscopy to evaluate PCI (Figure 5B). While the majority (64.2%) would not refer this case to gynae-oncology, over a quarter (25.9%) would refer a Krukenberg tumour to gynae-oncology for an oophorectomy (Figure 5C). Most surgeons (63.0%) agreed or strongly agreed with referring this case to a peritoneal centre for consideration of CRS and HIPEC (Figure 5D).

### Management of incidental synchronous peritoneal metastases

**Figure 6:**
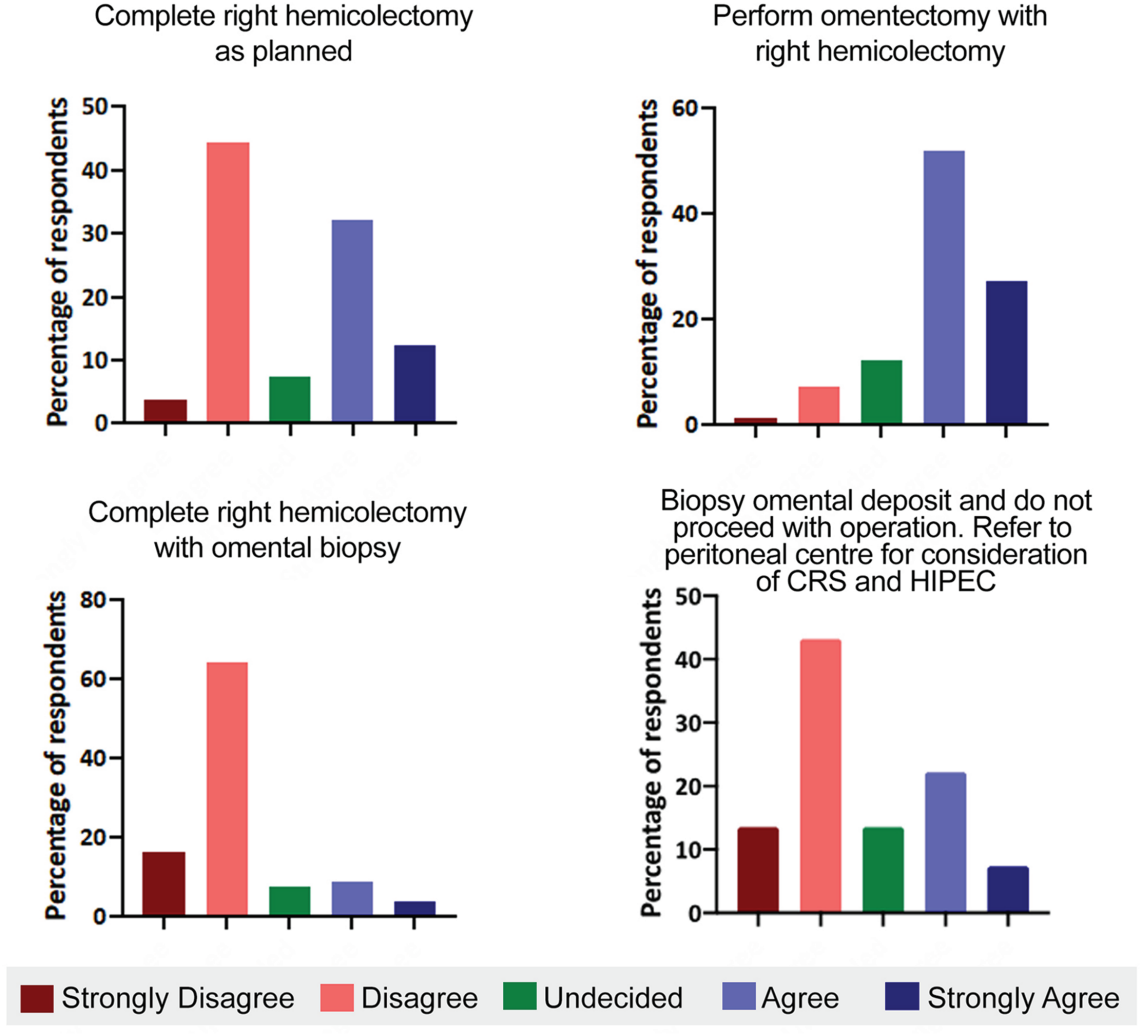
(A–D) Management options when incidental resectable synchronous omental metastases are found during a right hemicolectomy.

Question 9 evaluated the management strategy employed when resectable synchronous peritoneal metastases are incidentally encountered. The decision between proceeding with the right hemicolectomy or not was evenly divided with 46.7% of surgeons favouring completing surgery as planned, and approximately the same proportion not in favour of completing surgery as planned (Figure 6A). Most (79%) felt an omentectomy should be performed with right hemicolectomy to complete the surgery (Figure 6B). Most surgeons (77.8%) disagreed or strongly disagreed with performing the right hemicolectomy and merely taking a biopsy of the omental metastases (Figure 6C). Almost a third (29.6%) favoured only taking a biopsy of an omental deposit and referring the patient for consideration of CRS and HIPEC (Figure 6D).

### Risk factors for peritoneal recurrence

Question 10 evaluated surgeons’ perceptions on risk factors for peritoneal recurrence. Over three quarters (76.5%) felt a non-perforated T4 cancer conferred a very high or above average risk for peritoneal recurrence. Almost all surgeons felt that a perforated cancer (97.5%), ovarian metastases (97.5%) and isolated peritoneal metastases resected at index operation (100%) carried an above average or very high risk for peritoneal recurrence. Intra-operative tumour spillage was seen as an above average risk factor by two thirds (66.7%) of surgeons, with an obstructed tumour seen as average risk by the majority (54.3%).

### Role of surgical experience on management of peritoneal metastases

Of the 81 respondents, 55 (67.9%) had 5 or more years experience as a colorectal surgeon, with the remaining 26 (32.1%) having less than 5 years experience.

Two questions yielded significantly different responses based on surgical experience, with all other questions having similar responses regardless of surgical experience.

In evaluating the role of a pro-active ‘second look’ surgery and HIPEC 6 months after a T4a resection, younger surgeons agreed with performing a diagnostic laparoscopy to assess PCI compared to more experienced surgeons (38.5% vs. 9.1%, p=0.036) (Suppl. Figure 1A). Similarly, younger surgeons agreed to refer such a case to a peritoneal centre for consideration of ‘second look’ surgery and HIPEC compared to experienced surgeons (15.4% vs. 1.8%, p=0.014) (Suppl. Figure 1B).

## Discussion

To our knowledge, this study provides the first snapshot of perceptions among colorectal surgeons in Australia and New Zealand in the management of CRPM. CRS and HIPEC is a complex procedure, with major morbidity and mortality rates ranging from 13.1–47.2% and 1.0–4.1%, respectively [9, 14]. Numerous studies have demonstrated a consistent relationship between high volume centres and improved long term survival after cancer surgery [15, 16]. Furthermore, there is a clear learning curve of approximately 140–220 cases that has to be overcome before achieving proficiency [17, 18]. Therefore, having a centralised system for management of peritoneal disease ensures selected centres can develop and maintain a high level of expertise in managing peritoneal disease.

While a number of international consensus guidelines support the role of CRS and HIPEC in the treatment of isolated CRPM [19, 20], it must be noted that most of the data supportive of CRS and HIPEC is in the form of cohort studies that report a median survival of 30–58 months [7, 8, 9, 10] with a paucity of randomised data [4]. Furthermore, CRS and HIPEC are offered to selected cases, while systemic chemotherapy is offered to a more unselected cohort. Systemic chemotherapy can offer patients with CRPM a median survival of only 16 months [1], with a 5-year survival of less than 5% [21]. In ovarian cancer, a recent RCT [22] demonstrated that CRS and HIPEC offered a significantly improved recurrence free and overall survival compared to CRS with systemic therapy alone. This trial did however demonstrate poorer survival in both arms compared to previous trials in ovarian peritoneal disease [23]. It is plausible that this landmark trial may lead to greater consideration for CRS and HIPEC in advanced ovarian cancer [24].

While the superiority of CRS and HIPEC over systemic chemotherapy has been previously demonstrated in an RCT [4], the role of systemic chemotherapy as an adjunct to CRS and HIPEC remains unexplored. Various studies [6, 25] have reported an improved survival with the use of neoadjuvant or adjuvant systemic chemotherapy with CRS and HIPEC. However, based on current evidence, there is very limited role for systemic chemotherapy as mainstay of treatment for resectable isolated CRPM [26]. The role of systemic chemotherapy as an adjunct to CRS and HIPEC, in the neoadjuvant or adjuvant setting is currently under investigation in the CAIRO 6 trial (NCT02758951) [27].

While CRS with HIPEC is viewed as the mainstay of treatment for isolated CRPM, recent evidence has raised questions about the efficacy of HIPEC. The recently completed PRODIGE 7 trial [28] demonstrated an impressive 41.2 months median overall survival following CRS alone, with the addition of HIPEC not offering a significant survival benefit (41.2 months vs. 41.7 months, HR 1.00; 95% CI: 0.73–1.37). Subgroup analysis however demonstrated a survival benefit with HIPEC in those with a PCI 11–15. This trial, while yet unpublished, reaffirmed the value of complete cytoreduction, but has raised doubts about the overall efficacy of oxaliplatin-based HIPEC.

The role of pro-active “second look” surgery in high-risk cases for peritoneal recurrence is controversial. In approximately 25% of primary CRC resections, there are clinical or pathological findings that indicate a high risk for peritoneal recurrence [29]. Given well-established factors that predict early peritoneal recurrence such as T4a pathology, ovarian metastases or perforated cancers, studies have evaluated the role of a pro-active approach in patients at high risk for peritoneal recurrence. Elias et al. [30] demonstrated that asymptomatic peritoneal metastases were diagnosed in 55% of cases undergoing a second-look laparotomy 13 months after resection of a high-risk primary colorectal cancer. These included cases of synchronous peritoneal metastases, ovarian metastases or perforated primary tumours. In a follow up study [31], peritoneal metastases were found in 56% of patients undergoing second-look laparotomy for CRS and HIPEC 12 months following high-risk primary colorectal cancer resection. Following successful CRS and HIPEC at second-look surgery, 5-year OS was 90%, with a 44% DFS.

Recent RCTs however, offer a different view. The recently completed French RCT (Prophylochip-NCT01226394) [32] explored the role of systematic second-look surgery and oxaliplatin based HIPEC vs. surveillance for asymptomatic patients 6 months following a high risk primary colorectal cancer resection. High risk was defined as minimal CRPM resected with the primary, ovarian metastases or perforated primary tumour. While peritoneal metastases were diagnosed in 52% of patients undergoing second-look surgery, there was no difference in 3-year DFS (44% vs. 51%, p=0.75) or 3-year OS (79% vs. 80%) in the second look or surveillance arms. Similarly, the COLOPEC trial (NCT02231086) [33] evaluated the role of adjuvant oxaliplatin based HIPEC after pT4 or perforated primary colorectal cancers. There was no difference in the primary endpoint of peritoneal metastases free survival (77% vs. 81%) by diagnostic laparoscopy at 18 months in the control and adjuvant HIPEC arms. Furthermore, there was no difference in DFS and OS at 18 months between the two arms.

All these studies confirm the validity of high risk factors for peritoneal recurrence. However, based on current evidence, early re-look with oxaliplatin based HIPEC or adjuvant oxaliplatin based HIPEC may not offer any survival benefit over surveillance. These trials are yet unpublished, and the final publications may offer further insight into why the results were unfavourable.

Krukenberg tumours are ovarian metastases commonly from cancers such as gastric, colorectal or appendiceal. The presence of a new ovarian mass, an elevated CEA and a previous CRC, is a Krukenberg tumour until proven otherwise. Ovarian metastases from CRC are seen in 4–19% of cases [34]. They are often chemo-resistant, associated with other peritoneal metastases, and confer a worse prognosis [35, 36]. Ovarian metastases from CRC are peritoneal disease and therefore should be treated with CRS and HIPEC [37, 38]. A laparoscopy may be performed to evaluate PCI as other peritoneal deposits may be present.

Synchronous CRPM are seen in 4–13% of patients [39, 40, 41]. There are currently no guidelines to direct care in patients with incidentally found resectable synchronous CRPM. A recent Dutch study [42] comparing outcomes after synchronous or metachronous management of CRPM with CRS and HIPEC demonstrated no difference in OS (34 vs. 33 months, p=0.819). Other studies have similarly demonstrated no difference in survival between metachronous and synchronous treatment of CRPM with CRS and HIPEC [43, 44]. Shida et al. [45] recently demonstrated that a synchronous R0 resection for CRPM without HIPEC can offer a favourable median survival of 33 months and a 5-year survival of 28.7%. This is an area with only retrospective studies in the literature. If only low volume incidental CRPM are incidentally found, findings from PRODIGE 7 [28] would also suggest HIPEC may not add any survival benefit. CRS and HIPEC or alternatively ensuring an R0 resection without HIPEC both appear to be reasonable options supported by studies in the management of incidentally found synchronous CRPM.

One of the limitations inherent in all surveys is low response rate. However, our response rate of 36.2% is much higher that other similar international surveys in this field [12, 13, 46]. Furthermore, while most other surveys [12, 13, 47, 48] have assessed clinician views and awareness on effectiveness and safety of CRS and HIPEC, our survey and a recent Swiss survey [46] served to evaluate how clinicians view and manage commonly encountered cases with peritoneal disease, as timely, appropriate and evidence based care in such cases translates to better clinical outcomes. While this survey covered the use of HIPEC, it failed to evaluate the role of other intraperitoneal chemotherapy modalities such as early postoperative intraperitoneal chemotherapy (EPIC) or pressurised aerosolised chemotherapy (PIPAC). While EPIC and PIPAC are very infrequently used in Australia and New Zealand, it would have nonetheless been useful to evaluate its perceived role among colorectal surgeons. This survey also failed to capture general surgeons and medical oncologists. In Australia and New Zealand, CRCs are managed by both general and colorectal surgeons. In metropolitan centres with the majority of the population, they are managed largely by colorectal surgeons. It would have been challenging to capture gastrointestinal medical oncologists only, as medical oncologists not involved in colorectal cancer care would have been unlikely to respond, leading to a very low response rate. Furthermore, we believe that peritoneal disease is largely a surgical disease, therefore we elected to survey the most common surgical group involved in the care of these patients.

## Conclusions

This survey provides the first snapshot of management strategies undertaken by colorectal surgeons in the management of CRPM in Australia and New Zealand. It demonstrates that while most colorectal surgeons have a similar view in the management of CRPM, there are some circumstances that lead to different management strategies. Regular published updates and ongoing collaborations with peritoneal services would help ensure appropriate utilisation of CRS and HIPEC resources.

## References

[1] Franko J, Shi Q, Meyers JP, Maughan TS, Adams RA, Seymour MT, et al. Prognosis of patients with peritoneal metastatic colorectal cancer given systemic therapy: an analysis of individual patient data from prospective randomised trials from the Analysis and Research in Cancers of the Digestive System (ARCAD) database. Lancet Oncol 2016;17:1709–19. 10.1016/S1470-2045(16)30500-9 27743922

[2] Chu DZ, Lang NP, Thompson C, Osteen PK, Westbrook KC. Peritoneal carcinomatosis in nongynecologic malignancy. A prospective study of prognostic factors. Cancer 1989;63:364–7. 10.1002/1097-0142(19890115)63:2<364::aid-cncr2820630228>3.0.co;2-v 2910444

[3] Sadeghi B, Arvieux C, Glehen O, Beaujard AC, Rivoire M, Baulieux J, et al. Peritoneal carcinomatosis from non-gynecologic malignancies: results of the EVOCAPE 1 multicentric prospective study. Cancer 2000;88:358–63. 10.1002/(sici)1097-0142(20000115)88:2<358::aid-cncr16>3.0.co;2-o 10640968

[4] Verwaal V, van Ruth S, de Bree E, van Slooten G, van Tinteren H, Boot H, et al. Randomized trial of cytoreduction and hyperthermic intraperitoneal chemotherapy versus systemic chemotherapy and palliative surgery in patients with peritoneal carcinomatosis of colorectal cancer. J Clin Oncol 2003;21:3737–43. 10.1200/JCO.2003.04.187 14551293

[5] Banaste N, Rousset P, Mercier F, Rieussec C, Valette PJ, Glehen O, et al. Preoperative nutritional risk assessment in patients undergoing cytoreductive surgery plus hyperthermic intraperitoneal chemotherapy for colorectal carcinomatosis. Int J Hyperthermia 2018;34:589–94. 10.1080/02656736.2017.1371342 28828897

[6] Elias D, Gilly F, Boutitie F, Quenet F, Bereder JM, Mansvelt B, et al. Peritoneal colorectal carcinomatosis treated with surgery and perioperative intraperitoneal chemotherapy: retrospective analysis of 523 patients from a multicentric French study. J Clin Oncol 2010;28:63–8. 10.1200/JCO.2009.23.9285 19917863

[7] Elias D, Lefevre JH, Chevalier J, Brouquet A, Marchal F, Classe JM, et al. Complete cytoreductive surgery plus intraperitoneal chemohyperthermia with oxaliplatin for peritoneal carcinomatosis of colorectal origin. J Clin Oncol 2009;27:681–5. 10.1200/JCO.2008.19.7160 19103728

[8] Glehen O, Kwiatkowski F, Sugarbaker PH, Elias D, Levine EA, De Simone M, et al. Cytoreductive surgery combined with perioperative intraperitoneal chemotherapy for the management of peritoneal carcinomatosis from colorectal cancer: a multi-institutional study. J Clin Oncol 2004;22:3284–92. 10.1200/JCO.2004.10.012 15310771

[9] Quenet F, Goere D, Mehta S, Roca L, Dumont F, Heississen M, et al. Results of two bi-institutional prospective studies using intraperitoneal oxaliplatin with or without irinotecan during HIPEC after cytoreductive surgery for colorectal carcinomatosis. Ann Surg 2011;254:294–301. 10.1097/SLA.0b013e3182263933 21772129

[10] Chua T, Morris D, Esquivel J. Impact of the peritoneal surface disease severity score on survival in patients with colorectal cancer peritoneal carcinomatosis undergoing complete cytoreduction and hyperthermic intraperitoneal chemotherapy. Ann Surg Oncol 2010;17:1330–6. 10.1245/s10434-009-0866-x 20033321

[11] Prada-Villaverde A, Esquivel J, Lowy A, Markman M, Chua T, Pelz J, et al. The American society of peritoneal surface malignancies evaluation of HIPEC with mitomycin C versus oxaliplatin in 539 patients with colon cancer undergoing a complete cytoreductive surgery. J Surg Oncol 2014;110:779–85. 10.1002/jso.23728 25088304

[12] Bernaiche T, Emery E, Bijelic L. Practice patterns, attitudes, and knowledge among physicians regarding cytoreductive surgery and HIPEC for patients with peritoneal metastases. Pleura Peritoneum 2018;3:1–7. 10.1515/pp-2017-0025 PMC6405017 30911650

[13] Braam HJ, Boerma D, Wiezer MJ, Ramshorst B. Cytoreductive surgery and HIPEC in treatment of colorectal peritoneal carcinomatosis: experiment or standard care? A survey among oncologic surgeons and medical oncologists. Int J Clin Oncol 2015;20:928–34. 10.1007/s10147-015-0816-5 25788217

[14] Nikolic S, Dzodic R, Zegarac M, Djurisic I, Gavrilovic D, Vojinovic V, et al. Survival prognostic factors in patients with colorectal peritoneal carcinomatosis treated with cytoreductive surgery and intraoperative hyperthermic intraperitoneal chemotherapy: a single institution experience. J Buon 2014;19:66–74. 24659645

[15] Rajeev R, Klooster B, Turaga KK. Impact of surgical volume of centers on post-operative outcomes from cytoreductive surgery and hyperthermic intra-peritoneal chemoperfusion. J Gastrointest Oncol 2016;7:122–8. 10.3978/j.issn.2078-6891.2015.099 PMC4754306 26941990

[16] van Gijn W, Gooiker GA, Wouters MW, Post PN, Tollenaar RA, van de Velde CJ. Volume and outcome in colorectal cancer surgery. Eur J Surg Oncol 2010;36:S55–63. 10.1016/j.ejso.2010.06.027 20615649

[17] Kusamura S, Baratti D, Deraco M. Mutidimensional analysis of the learning curve for cytoreductive surgery and hyperthermic intraperitoneal chemotherapy in peritoneal surface malignancies. Ann Surg 2012;255:348–56. 10.1097/SLA.0b013e3182436c28 22202584

[18] Yan TD, Links M, Fransi S, Jacques T, Black D, Saunders V, et al. Learning curve for cytoreductive surgery and perioperative intraperitoneal chemotherapy for peritoneal surface malignancy – a journey to becoming a nationally funded peritonectomy center. Ann Surg Oncol 2007;14:2270–80. 10.1245/s10434-007-9406-8 17464543

[19] Van Cutsem E, Cervantes A, Adam R, Sobrero A, Van Krieken JH, Aderka D, et al. ESMO consensus guidelines for the management of patients with metastatic colorectal cancer. Ann Oncol 2016;27:1386–422. 10.1093/annonc/mdw235 27380959

[20] Vogel JD, Eskicioglu C, Weiser MR, Feingold DL, Steele SR. The American society of colon and rectal surgeons clinical practice guidelines for the treatment of colon cancer. Dis Colon Rectum 2017;60:999–1017. 10.1097/DCR.0000000000000926 28891842

[21] Franko J, Shi Q, Goldman C, Pockaj B, Nelson G, Goldberg R, et al. Treatment of colorectal peritoneal carcinomatosis with systemic chemotherapy: a pooled analysis of north central cancer treatment group phase III trials N9741 and N9841. J Clin Oncol 2012;30:263–7. 10.1200/JCO.2011.37.1039 PMC3269953 22162570

[22] van Driel WJ, Koole SN, Sikorska K, Schagen van Leeuwen JH, Schreuder HW, Hermans RH, et al. Hyperthermic intraperitoneal chemotherapy in ovarian cancer. N Engl J Med 2018;378:230–40. 10.1056/NEJMoa1708618 29342393

[23] Armstrong DK, Bundy B, Wenzel L, Huang HQ, Baergen R, Lele S, et al. Intraperitoneal cisplatin and paclitaxel in ovarian cancer. N Engl J Med 2006;354:34–43. 10.1056/NEJMoa052985 16394300

[24] Farrell R. Is peritonectomy and hyperthermic intraperitoneal chemotherapy a new standard of treatment for advanced epithelial ovarian cancer? Aust NZ J Obstet Gynaecol 2019;59:335–40. 10.1111/ajo.12953 30706448

[25] Ceelen W, Nieuwenhove Y, Putte D, Pattyn P. Neoadjuvant chemotherapy with bevacizumab may improve outcome after cytoreduction and hyperthermic intraperitoneal chemoperfusion (HIPEC) for colorectal carcinomatosis. Ann Surg Oncol 2014;21:3023–8. 10.1245/s10434-014-3713-7 24756812

[26] Waite K, Youssef H. The role of neoadjuvant and adjuvant systemic chemotherapy with cytoreductive surgery and heated intraperitoneal chemotherapy for colorectal peritoneal metastases: a systematic review. Ann Surg Oncol 2017;24:705–20. 10.1245/s10434-016-5712-3 28058545

[27] Rovers KP, Bakkers C, Simkens G, Burger JW, Nienhuijs SW, Creemers GM, et al. Perioperative systemic therapy and cytoreductive surgery with HIPEC versus upfront cytoreductive surgery with HIPEC alone for isolated resectable colorectal peritoneal metastases: protocol of a multicentre, open-label, parallel-group, phase II–III, randomised, superiority study (CAIRO6). BMC Cancer 2019;19:390. 10.1186/s12885-019-5545-0 PMC6485075 31023318

[28] Quenet F, Elias D, Roca L, Goere D, Ghouti L, Pocard M, et al. A UNICANCER phase III trial of hyperthermic intra-peritoneal chemotherapy (HIPEC) for colorectal peritoneal carcinomatosis (PC): PRODIGE 7. J Clin Oncol 2018;36:LBA3503.

[29] Honoré C, Gelli M, Francoual J, Benhaim L, Elias D, Goéré D. Ninety percent of the adverse outcomes occur in 10% of patients: can we identify the populations at high risk of developing peritoneal metastases after curative surgery for colorectal cancer? Int J Hyperthermia 2017;33:505–10. 10.1080/02656736.2017.1306119 28540831

[30] Elias D, Goere D, Di Pietrantonio D, Boige V, Malka D, Kohneh-Shahri N, et al. Results of systematic second-look surgery in patients at high risk of developing colorectal peritoneal carcinomatosis. Ann Surg 2008;247:445–50. 10.1097/SLA.0b013e31815f0113 18376188

[31] Elias D, Honore C, Dumont F, Ducreux M, Boige V, Malka D, et al. Results of systematic second-look surgery plus HIPEC in asymptomatic patients presenting a high risk of developing colorectal peritoneal carcinomatosis. Ann Surg 2011;254:289–93. 10.1097/SLA.0b013e31822638f6 21709543

[32] Goere D, Glehen O, Quenet F, Ducreux M, Guilloit J, Texier M, et al. Results of a randomized phase 3 study evaluating the potential benefit of a second-look surgery plus HIPEC in patients at high risk of developing colorectal peritoneal metastases (PROPHYLOCHIP-NTC01226394). J Clin Oncol 2018;36:3531.

[33] Klaver C, Wisselink D, Punt C, Snaebjornsson P, Crezee J, Aalbers A, et al. Adjuvant HIPEC in patients with colon cancer at high risk of peritoneal metastases: primary outcome of the COLOPEC multicenter randomized trial. J Clin Oncol 2019;37:482.

[34] Koppe MJ, Boerman OC, Oyen WJ, Bleichrodt RP. Peritoneal carcinomatosis of colorectal origin: incidence and current treatment strategies. Ann Surg 2006;243:212–22. 10.1097/01.sla.0000197702.46394.16 PMC1448921 16432354

[35] Kuijpers AM, Mehta AM, Aalbers AG, van Driel WJ, Boot H, Verwaal VJ. Treatment of ovarian metastases of colorectal and appendiceal carcinoma in the era of cytoreductive surgery and hyperthermic intraperitoneal chemotherapy. Eur J Surg Oncol 2014;40:937–42. 10.1016/j.ejso.2014.02.238 24630923

[36] Evers DJ, Verwaal VJ. Indication for oophorectomy during cytoreduction for intraperitoneal metastatic spread of colorectal or appendiceal origin. Br J Surg 2011;98:287–92. 10.1002/bjs.7303 21046680

[37] Sugarbaker P, Averbach A. Krukenberg syndrome as a natural manifestation of tumor cell entrapment. Cancer Treat Res 1996;82:163–91. 10.1007/978-1-4613-1247-5_11 8849950

[38] Bushati M, Rovers KP, Sommariva A, Sugarbaker PH, Morris DL, Yonemura Y, et al. The current practice of cytoreductive surgery and HIPEC for colorectal peritoneal metastases: results of a worldwide web-based survey of the Peritoneal Surface Oncology Group International (PSOGI). Eur J Surg Oncol 2018;44:1942–8. 10.1016/j.ejso.2018.07.003 30075978

[39] Jayne D, Fook S, Loi C, Seow-Choen F. Peritoneal carcinomatosis from colorectal cancer. Br J Surg 2002;89:1545–50. 10.1046/j.1365-2168.2002.02274.x 12445064

[40] Lemmens V, Klaver Y, Verwaal V, Rutten H, Coebergh J, de Hingh I. Predictors and survival of synchronous peritoneal carcinomatosis of colorectal origin: a population-based study. Int J Cancer 2011;128:2717–25. 10.1002/ijc.25596 20715167

[41] Segelman J, Granath F, Holm T, Machado M, Mahteme H, Martling A. Incidence, prevalence and risk factors for peritoneal carcinomatosis from colorectal cancer. Br J Surg 2012;99:699–705. 10.1002/bjs.8679 22287157

[42] Hentzen J, Rovers KP, Kuipers H, van der Plas WY, Been LB, Hoogwater FJ, et al. Impact of synchronous versus metachronous onset of colorectal peritoneal metastases on survival outcomes after cytoreductive surgery (CRS) with hyperthermic intraperitoneal chemotherapy (HIPEC): a multicenter, retrospective, observational study. Ann Surg Oncol 2019;26:2210–21. 10.1245/s10434-019-07294-y PMC6545176 30877495

[43] Maillet M, Glehen O, Lambert J, Goere D, Pocard M, Msika S, et al. Early postoperative chemotherapy after complete cytoreduction and hyperthermic intraperitoneal chemotherapy for isolated peritoneal carcinomatosis of colon cancer: a multicenter study. Ann Surg Oncol 2016;23:863–9. 10.1245/s10434-015-4914-4 26480848

[44] Rivard J, McConnell Y, Temple W, Mack L. Cytoreduction and heated intraperitoneal chemotherapy for colorectal cancer: are we excluding patients who may benefit? J Surg Oncol 2014;109:104–9. 10.1002/jso.23446 24449172

[45] Shida D, Tsukamoto S, Ochiai H, Kanemitsu Y. Long-term outcomes after R0 resection of synchronous peritoneal metastasis from colorectal cancer without cytoreductive surgery or hyperthermic intraperitoneal chemotherapy. Ann Surg Oncol 2018;25:173–8. 10.1245/s10434-017-6133-7 29063295

[46] Grass F, Martin D, Montemurro M, Mathevet P, Wolfer A, Coukos G, et al. Current opinion and knowledge on peritoneal carcinomatosis: a survey among a Swiss oncology network. Chemother 2018;63:143–7. 10.1159/000488774 29898438

[47] Yoo HJ, Hong JJ, Ko YB, Lee M, Kim Y, Han HY, et al. Current practices of cytoreductive surgery and hyperthermic intraperitoneal chemotherapy in the treatment of peritoneal surface malignancies: an international survey of oncologic surgeons. World J Surg Oncol 2018;16:92. 10.1186/s12957-018-1377-7 PMC5952844 29764445

[48] Spiegle G, Schmocker S, Huang H, Victor JC, Law C, McCart JA, et al. Physicians’ awareness of cytoreductive surgery and hyperthermic intraperitoneal chemotherapy for colorectal cancer carcinomatosis. Can J Surg 2013;56:237–42. 10.1503/cjs.003912 PMC3728242 23883493

